# Incidence and features of maxillofacial fractures at Jordanian tertiary hospital before, during and after the COVID-19 period

**DOI:** 10.4317/medoral.25835

**Published:** 2023-01-15

**Authors:** Anwar B Bataineh

**Affiliations:** 1 BDS, MScD, MDSc, CSOS. Professor of Oral and Maxillofacial Surgery, Faculty of Dentistry, Jordan University of Science and Technology, Irbid, Jordan

## Abstract

**Background:**

The purpose of this retrospective cohort study is to investigate the incidence and treatment of maxillofacial fractures before, during, and after the COVID-19 pandemic.

**Material and Methods:**

This single-center study was conducted at King Abdullah University Hospital (KAUH). The required data was obtained from the electronic clinical records of all patients in whom maxillofacial fractures were confirmed by plain radiography and computed tomography (CT) between January 2019 and December 2021, allowing for a 12-month period before, during and after the COVID-19 pandemic.

**Results:**

During the study period, 595 maxillofacial fractures in 311 patients (234 males and 77 females, mean age 27.28 years) were treated. The most frequent affected age was 21-30 years old in the before and after COvid- 19 period (92 patients, 29.58.%), while in during-COVID-19 period it was 11-20 years old (22 patients, 7.07%). There was similarity in male predominance, RTA cause, anatomical site was the mandible, the type anatomical complexity was single, treatment procedure was ORIF in all three periods.

**Conclusions:**

The incidence of maxillofacial fractures during the COVID-19 pandemic period was lower compared to the periods before and after the pandemic. Given that most fractures were caused by RTAs, these findings are expected, as movement was restricted during lockdown.

** Key words:**Incidence, features, maxillofacial fracture, trauma, COVID-19, Jordan.

## Introduction

As the COVID-19 infection started to spread across the globe in early 2020, the governments of most countries started implementing severe movement restrictions in order to protect public health and help the health system cope with the surge in patient numbers (1). These measures had a profound effect on traumatology, as fracture epidemiology primarily depends on human behavior and lifestyle (2,3). On March 11, 2020, the World Health Organization officially declared the COVID-19 pandemic (4), with adoption of social lockdowns across the globe (5). The Jordanian government declared a state of emergency on March 19, 2020 and imposed a curfew on March 21, 2020.

The data yielded by this rapidly developing field of research indicate that maxillofacial fractures are increasing in frequency and severity, due the heavy dependence on road transportation (6,7), resulting in a severe burden on the emergency rooms. However, these issues were further exacerbated during the COVID-19 pandemic, as almost all surgical procedures involving maxillofacial fractures require intimate contact with the naso-oro-pharyngeal region which carries a high viral load if the patient is positive for COVID-19 (8). Thus, it is surprising that the effects of lockdown on the epidemiology of maxillofacial fractures remain insufficiently investigated.

This gap in the extent literature motivated the present retrospective cohort study, the aim of which was to investigate the incidence and treatment of maxillofacial fractures before, during, and after the COVID-19 pandemic.

Materials and Methods

This single-center retrospective cohort study was conducted at the Department of Oral and Maxillofacial Surgery of King Abdullah University Hospital (KAUH) in Irbid, northern Jordan.

Due to the retrospective nature of this study, it was granted an exemption by the Jordan University of Science and Technology IRB, while the need for informed consent was waived because no patient involvement was required. This study followed the Declaration of Helsinki on medical protocol and ethics and the regional Ethical Review Board.

The electronic clinical records of all patients in whom maxillofacial fractures were confirmed via plain radiography and computed tomography (CT) between January 2019 and December 2021 were retrospectively retrieved from the central database registry of the hospital where they were treated at the Department of Oral and Maxillofacial Surgery. During the social lockdown period, all hospital admissions were preceded by telephonic triage to ascertain if patients exhibit any signs or symptoms suggestive of COVID-19 infection. All admitted patients were managed following the latest guidelines to reduce the risk of infection for the healthcare workers.

As the aim of this study was to investigate maxillofacial fracture incidence and type across three periods, 12-month intervals were allocated for each one, whereby the period before the COVID-19 pandemic spanned from January 1, 2019 to December 31, 2019, the COVID-19 period spanned from January 1, 2020 to December 31, 2020, and the period after the COVID-19 pandemic spanned from January 1, 2021 to December 31, 2021.

Inclusion criteria: Patients were included if they had undergone surgery for a maxillofacial fracture in any of the three time periods defined above. Exclusion criteria: Patients were excluded from the analyses if their medical records were incomplete, they had a history of previous maxillofacial fractures, pathological fractures, or they suffered from isolated dental trauma.

The data retrieved from the patient records included age, sex, date of injury, cause of injury, maxillofacial fracture type, anatomical complexity of maxillofacial fractures, and treatment mode. The injury causes were classified as road traffic accident (RTA), assault, falls, industrial accident, sports, or gunshots. Maxillofacial fractures included mandibular, maxillary, zygomatic, and orbital fractures. When patients had multiple fractures in different facial bones, each fracture was registered separately. Mandibular fractures were classified as dentoalveolar, symphysis, parasymphysis, body, ramus, angle, or condyle fractures. Maxillary fractures were classified as dentoalveolar, LeFort I, LeFort II, or LeFort III. Zygomatic fractures were classified as zygomaticomaxillary complex, zygomatic arch, zygomaticomaxillary suture (ZMS), or zygomaticofrontal suture (ZFS) fractures. Orbital fractures were classified as floor, lateral wall, medial wall, or roof fractures. Moreover, maxillofacial fractures were classified by type as simple, comminuted, compound, or greenstick, and their anatomical complexity was classified as single, double, or multiple fractures. Finally, treatment was classified as Open Reduction Internal Fixation (ORIF), closed reduction, or conservative. The primary study outcome was the incidence of maxillofacial fractures, while the types of maxillofacial fractures and treatment mode were considered secondary outcomes.

Data were analyzed using IBM SPSS version 28.0. Categorical data were presented as frequencies and percentages and Chi-square test was conducted to assess the associations between independent variables, whereby *p* ≤ 0.05 was considered statistically significant.

## Results

During the study period, 595 maxillofacial fractures in 311 patients (234 males and 77 females, mean age 27.28 years) were treated at the Department of Oral and Maxillofacial Surgery. Most maxillofacial fractures occurred in patients aged 21-30 years and were primarily of the simple type (152 patients, 48.9%), caused by RTA (61.09% of the patients). The most frequent anatomical site was the mandible (101 cases, 39.76%), and most patients suffered simple maxillofacial fractures (152, 48.9%), which were largely single fractures (139 patients, 44.7%). Finally, the most commonly applied treatment mode was ORIF (249 patients, 80.1%). No statistical analysis was performed on the differences in the anatomical site of maxillofacial fractures across the three time periods (before, during, and after COVID-19 pandemic), because patients had multiple maxillofacial fractures.

- The period before the COVID-19 pandemic

During 2019, before the pandemic, 137 (44.05%) patients with 254 (42.69%) maxillofacial fractures (103 males and 34 females) were treated at our clinic. The greatest number of patients was in the 21-30 age group (48 cases, 35.04%), while those in the > 50 group were the least represented (9 patients, 6.57%). As shown in Table 1 and Table 2, respectively, the most frequently encountered etiology was RTA (88 patients, 64.23%) and the most frequent anatomical site was the mandible (101 cases, 39.76%), followed by orbit (68 cases, 26.77%). Most maxillofacial fractures were simple (70 patients, 51.09%) and involved single fractures (64 patients, 46.72%), as shown in Table 3. Finally, a large majority of patients (112, 81.75%) required ORIF (Table 4).

**Table 1:**
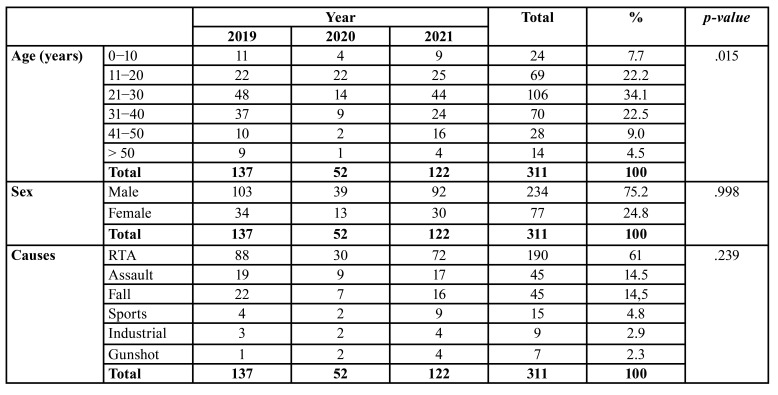
Distribution of fractures according to age, sex and causes before, during and after COVID-19

**Table 2:**
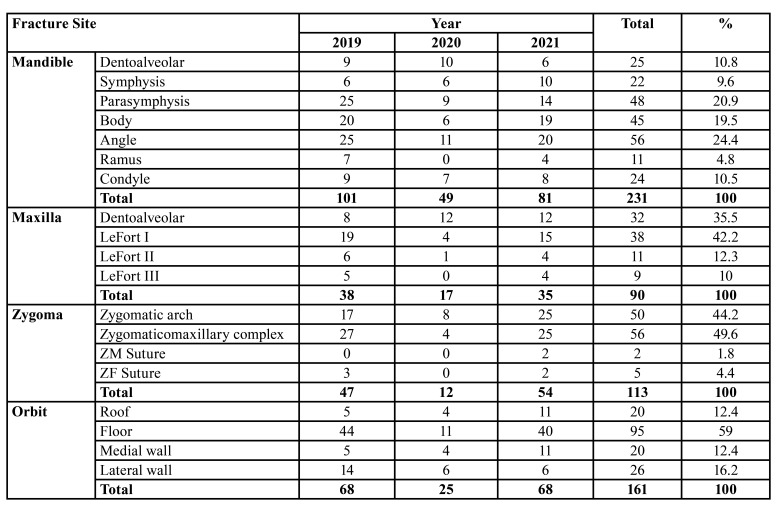
Distribution of maxillofacial fractures according to anatomical site before, during and after COVID-19.

**Table 3:**
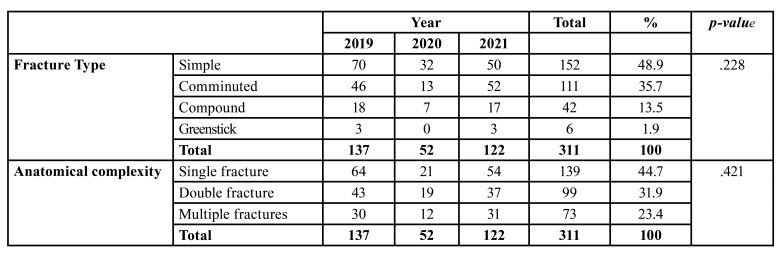
Distribution of maxillofacial fractures according to type and anatomical complexity before, during and after COVID-19.

**Table 4:**
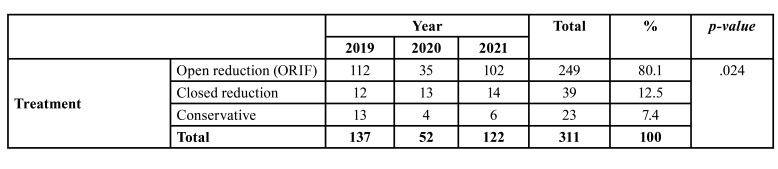
Distribution of maxillofacial fractures according to treatment mode before, during and after COVID-19.

- The COVID-19 period

During the pandemic, 52 (16.72%) patients with 103 (17.31%) maxillofacial fractures (39 males and 13 females) were treated at our clinic. The greatest number of patients was in the 21-30 age group (22 cases, 42.30%), while only one individual was aged > 50 (1.92%). As shown in Table 1 and Table 2 respectively, the most common etiology was RTA (30 patients, 57.69%) and the most prevalent anatomical site was the mandible (49 cases, 19.29%), followed by orbit (25 cases, 9.84%). Most maxillofacial fractures were simple (32 patients, 61.54%) and involved single fractures (21 patients, 40.38%), as shown in Table 3. Finally, as indicated in Table 4, most patients (35, 67.30%) required ORIF.

- The period after the COVID-19 pandemic

During 2021, 122 (39.23%) patients with 238 (40%) maxillofacial fractures (92 males and 30 females) were treated in our department. Again, those in the 21-30 age group predominated (44 cases, 36.07%), while those in the > 50 group were least represented (4 patients, 3.28%). As before, RTA was the most frequently encountered etiology (72 patients, 59.07%) and the mandible was the most frequent anatomical site (81 cases, 31.89%), followed by orbit (68 cases, 26.77%). In this period, however, most maxillofacial fractures were comminuted (52 patients, 42.62%) and involved single fractures (54 patients, 44.26%), as shown in Table 3. Finally, as can be seen from Table 4, a large majority of patients (102, 83.60%) required ORIF.

## Discussion

The present study was conducted at the King Abdullah University Hospital (KAUH), which is the largest and only tertiary hospital in northern Jordan, an area served by a further eleven hospitals (four of which are under the Ministry of Health, four are private, and three are military hospitals). This healthcare institution provides care to directly admitted patients as well as those that are transferred from peripheral hospitals due to more severe and complex trauma. As a result, the data used in the present study do not include all trauma cases in our region that occurred during the 2019-2021 period. As can be seen from the current analyses, during the COVID-19 lockdown, a decline in maxillofacial fractures (up to 40%) was noted compared to 2019 and 2021. This reduction was primarily attributed to the decrease in road traffic accidents, as the numbers of patients with other forms of trauma remained relatively unchanged. These findings are expected, as mobility was severely restricted during lockdown, resulting in less traffic on the roads. The Jordanian authorities also ordered all citizens to stay in their homes, banned travel between provinces, as well as temporarily closed all grocery stores and pharmacies. However, even after lockdown, the Jordanian government strongly advised that can to continue to work from home and limit travel.

Nonetheless, the number of patients seen at our department was the lowest between March 2020 and December 2020. However, as the incidence of maxillofacial fractures tends to vary considerably according to geographic region, socioeconomic status, culture, religion, and population demographics, some changes in their etiological distribution and other characteristics were expected during the COVID-19 outbreak (9,10). Concurring with the analyses of other studies performed, as did not indicate an increase in interpersonal or domestic violence during lockdown (11). Regarding the patient age, as in other studies of this type, most of the patients seen at our department were in the 21-30 age category (mean age 27.28 years) (12,13). Likewise, the majority of patients were male, male/female ratio (3.03:1), albeit at a somewhat lower incidence of maxillofacial fractures in females, than that reported by other authors, ranging from 5.2:1 to 8:1:1.4 (14-16).

As expected, and in line with other studies, most maxillofacial fractures treated at our department were caused by RTAs (6,17-20). While RTAs are generally more prevalent in developing compared to developed countries, the current findings are also attributed to the weak road infrastructure, as well as disregard for traffic rules and safety laws. According to the official statistics provided by the Jordan Public Security Directorate, during 2019, 10,875 traffic accidents with 17,013 injuries were recorded, which corresponded to 29.7 accidents and 46.6 persons injured per day. During 2020, the number of traffic accidents declined to 8,451, with a corresponding reduction in injuries [12,690], which is equivalent to 23.2 accidents and 34.8 persons injured per day. During 2021, 589 traffic accidents with 17,485 injuries were registered (or 8.30 accidents and 47.9 injuries per day).

However, during the COVID-19 pandemic, the epidemiology of maxillofacial fractures due to traffic accidents also changed. Specifically, mandibular fractures were the most common, concurring with the results obtained in other countries (21-24). The orbital bone was the second most common fracture site, consistent with the other reported findings (25), while differing from the observations made by other authors, who noted the highest frequency of zygomatic and mandibular fractures, most of which were noted in males (26,27), even though in extant literature women are cited as more frequent assault victims (28).

During the COVID-19 pandemic, all patients with maxillofacial fractures were given preoperative PCR tests to avoid spread of infection even if the patients were asymptomatic. In the periods before and after the COVID-19 pandemic, while the number of patients treated for maxillofacial fractures was greater than during the pandemic, other features were comparable, except for comminuted (rather than simple) fractures which predominated in the after-pandemic period. The current findings also coincide with those reported by other authors, as they indicate that ORIF combined with internal fixation was the primary treatment modality irrespective of the analyzed period, and in a few cases (7.4%) maxillofacial fractures were treated conservatively (29,30).

## Conclusions

As expected, given that mobility restrictions during lockdown resulted in fewer traffic accidents, this led to fewer maxillofacial fractures treated at our department. Therefore, it is essential to implement more stringent road safety rules in order to allow these benefits to continue even after the lockdown.
